# Increased body mass index and vein diameter are associated with incomplete target vein closure following microfoam ablation of incompetent saphenous veins

**DOI:** 10.1016/j.jvsv.2023.09.004

**Published:** 2023-10-01

**Authors:** Stephanie D. Talutis, Amanda L. Chin, Peter F. Lawrence, Karen Woo, Steven M. Farley, William Duong, Juan Carlos Jimenez

**Affiliations:** Gonda Venous Center, Division of Vascular and Endovascular Surgery, David Geffen School of Medicine at UCLA, Los Angeles, CA

**Keywords:** Body mass index, Microfoam, Obesity, Saphenous, Varicose vein

## Abstract

**Objective:**

Patient characteristics and risk factors for incomplete or non-closure following thermal saphenous vein ablation have been reported. However, similar findings have not been clearly described following commercially manufactured polidocanol microfoam ablation (MFA). The objective of our study is to identify predictive factors and outcomes associated with non-closure following MFA of symptomatic, refluxing saphenous veins.

**Methods:**

A retrospective review of a prospectively maintained patient database was performed from procedures in our Ambulatory Procedure Unit. All consecutive patients who underwent MFA with commercially manufactured 1% polidocanol microfoam for symptomatic superficial vein reflux between June 2018 and September 2022 were identified. Patients treated for tributary veins only, without truncal vein ablation, were excluded. Patients were then stratified into groups: complete closure (Group I) and non-closure (Group II). Preoperative demographics, procedural details, and postoperative outcomes were analyzed. Preoperative variables that were significant on univariate analysis (prior deep venous thrombosis [DVT], body mass index [BMI] ≥30 kg/m^2^, and vein diameter) were entered into a multivariate logistic regression model with the primary outcome being vein non-closure.

**Results:**

Between June 2018 and September 2022, a total of 224 limbs underwent MFA in our ambulatory venous center. Of these, 127 limbs in 103 patients met study inclusion criteria. Truncal veins treated included the above-knee great saphenous vein (Group I: n = 89, 77% vs Group II: n = 7, 58%; *P* = .14), below-knee great saphenous vein (Group I: n = 7, 6% vs Group II: n = 0; *P* = .38), anterior accessory saphenous vein (Group I: n = 17, 15% vs Group II: n = 4, 33%; *P* = .12, and small saphenous vein (Group I: n = 4, 4% vs Group II: n = 1, 8%; *P* = .41). Complete closure (Group I) occurred in 115 limbs, and 12 limbs did not close (Group II) based on postoperative duplex ultrasound screening. The mean BMI in Group II (36.1 ± 6.4 kg/m^2^) was significantly greater than Group I (28.6 ± 6.1 kg/m^2^) (*P* < .001). Vein diameter of ≥10.2 mm was independently associated with truncal vein non-closure with an odds ratio of 4.8. The overall mean foam volume was 6.2 ± 2.7 ml and not different between the two cohorts (Group I: 6.2 ± 2.6 ml vs Group II: 6.3 + 3.5 ml; *P* = .89). Post MFA improvement in symptoms was higher in Group I (96.9%) compared with Group II (66.7%) (*P* = .001). The mean postoperative Venous Clinical Severity Score was also lower in Group I (8.0 ± 3.0) compared with Group II (9.9 ± 4.2) (*P* = .048). The overall incidences of ablation-related thrombus extension and DVT were 4.7% (n = 6) and 1.6% (n = 2), and all occurred in Group I. All were asymptomatic and resolved with anticoagulation.

**Conclusions:**

Microfoam ablation of symptomatic, refluxing truncal veins results in excellent overall closure rates and symptomatic relief. BMI ≥30 kg/m^2^ and increased vein diameter are associated with an increased risk of saphenous vein non-closure following MFA. Non-closure is associated with less symptomatic improvement and a lower post-procedure reduction in Venous Clinical Severity Score. Despite the incidence of ablation-related thrombus extension and DVT in this study being higher than reported rates following thermal ablation, MFA is safe for patients with early postoperative duplex ultrasound surveillance and selective short-term anticoagulation.

**Article Highlights**

• **Type of Research:** Single-center retrospective cohort study
• **Key Findings:** Body mass index >30 kg/m^2^ and truncal vein diameter >10.2 mm were associated with non-closure following treatment of 127 limbs in 103 patients with commercially manufactured polidocanol microfoam.
• **Take Home Message:** Body mass index >30 kg/m^2^ and vein diameter >10.2 mm are associated with non-closure following microfoam ablation of refluxing truncal veins.

Although thermal truncal vein closure is associated with high initial and long-term success, there are recognized patient characteristics associated with incomplete or non-closure. There is also supportive evidence that non-closure is predictive of inferior clinical outcomes.^1^ Two reported risk factors following thermal ablation include obesity and increased truncal vein diameter, but each varies based upon the method of ablation.^2^ Similar correlations between these variables and truncal polidocanol microfoam ablation (MFA) (Varithena, Boston Scientific) have not yet been clearly established.^3^^,^^4^ The objective of our study is to identify predictive factors and outcomes associated with non-closure following MFA of symptomatic, refluxing saphenous veins.

## Methods

A review of a prospectively maintained patient database was performed from procedures in our Ambulatory Procedure Unit. Approval was obtained from our institutional review board for the implementation and publication of this study; the requirement for patient consent for participation in the study was waived by our institutional review board based on the policy for minimal risk research. Our Ambulatory Procedure Unit is affiliated with a large, metropolitan tertiary care academic medical center. All consecutive patients who underwent MFA with commercially manufactured 1% polidocanol microfoam for symptomatic superficial vein reflux between June 2018 and September 2022 were identified. Patients treated for tributary veins only, without truncal vein ablation, were excluded. Additional exclusion criteria included: patients who were classified as CEAP clinical class 0 and 1 and those who did not undergo at least one post-procedure ultrasound and follow-up visit.

Symptomatic patients were defined as those who presented with lifestyle-limiting symptoms and physical findings of chronic venous disease. These include but are not limited to pain, aching, heaviness, fatigue, itching, edema, lipodermatosclerosis, and both active and healed ulceration. Duplex ultrasound evidence of dilated truncal veins ≥3.5 mm and reflux ≥0.5 seconds occurring at the saphenofemoral (SFJ) and/or saphenopopliteal junction (SPJ) and in the caudal length in the upright position was required for study inclusion. Prior to MFA, all patients had failed conservative therapy with at least 6 weeks of compression therapy (minimum below-knee compression stockings, 20-30 mmHg). All patients with venous ulcers were treated prior to MFA and concurrently in our wound care center and were compliant with weekly dressing changes, debridement, offloading, nutritional counseling, and multilayer compression.

We performed MFA of the great (GSV), anterior accessory (AASV), and small saphenous veins (SSV) according to previously published techniques and in accordance with manufacturer’s instructions for use.^5^^,^^6^ All MFA procedures were performed in our ambulatory venous center under local anesthesia. Ultrasound-guided venous access was obtained with either a 4F micropuncture needle and 4F sheath or with a 21 g butterfly needle. Following sheath or needle placement, the limb was elevated to greater than 45°. Prior to Varithena injection, 10 cc of sterile saline were injected retrograde into the treated veins while in the elevated position to greater than 45° followed by the Varithena injection. Microfoam volume was limited to ≤15 ml per session and was usually <5 ml in a single site. Large perforator veins were identified during preoperative and intraoperative vein mapping, and occlusive digital pressure was held over them during microfoam injection. For truncal veins, pressure was held 5 cm caudal to either the SFJ or SPJ using the ultrasound transducer for 5 minutes. During this time, the patient was instructed to dorsiflex and plantar flex the ankle for 20 repetitions. Immediately following the procedure, the femoral and popliteal veins were evaluated for acute thrombus with intraoperative ultrasound, and compressibility was assessed. The treated limb was compressed in the elevated position (>45°) with abdominal pads overlying the treated veins, using long-stretch bandages.

All patients in this study had a postoperative duplex (48-72 hours) ultrasound (PDUS) performed to assess for target vein closure, ablation-related thrombus extension (ARTE), and deep venous thrombosis (DVT). Patients were instructed to wear 20- to 30-mmHg thigh-high compression stockings for 14 days after their procedure as frequently as tolerated. Clinical follow up was performed by a vascular surgeon 3 to 6 weeks post-procedure.

After the initial PDUS, routine surveillance imaging was not performed unless the patient presented with leg symptoms (ie, persistent pain, redness, tenderness, swelling, ulceration, superficial phlebitis) or physical signs of incomplete vein ablation. The rationale for this protocol is that we do not recommend invasive endovenous treatment for superficial venous reflux or recanalization if they are asymptomatic. Postoperative leg pain and phlebitis (defined as redness and or tenderness at the site of a thrombosed vein) were evaluated clinically by the vascular surgeon who performed the procedure and monitored at each postoperative visit.

Successful closure was defined as absence of venous flow within 5 cm from the SFJ or SPJ. Non-closure was defined when persistent venous flow was present for >5 cm from the SFJ or SPJ. Patients were then stratified into groups: complete closure (Group I) and non-closure (Group II). Preoperative variables were analyzed between these two cohorts. These included demographics, body mass index (BMI), truncal vein diameters, history of DVT, oral anticoagulation therapy at the time of ablation, the presence of deep venous reflux, superficial reflux times, and baseline CEAP Clinical class and Venous Clinical Severity Scores (VCSS). These variables were chosen because they are associated with impaired clinical outcomes following thermal ablation (ie, laser and radiofrquency ablation [RFA]). We hypothesized that these variables may similarly impact saphenous vein non-closure following MFA. Procedural details analyzed included specific truncal vein treated, truncal and tributary vein diameters, volume of microfoam used, tributary vein sclerotherapy, tributary vein phlebectomy, and operative times. Postoperative outcomes analyzed included vein closure, symptomatic improvement, VCSS, ulcer healing rates, and thrombotic and non-thrombotic complications.

### Statistical analysis

Differences between Groups I (complete closure) and II (non-closure) were evaluated using the χ^2^ test for categorical variables and the Student *t*-test for continuous variables. Statistical significance was defined as *P* < .05. The variables that were significant on univariate analysis were then entered into a multivariate logistic regression model. Variables included were prior DVT, BMI ≥30 kg/m^2^, and vein diameter ≥10.2 mm. The vein diameter used represented the 75th percentile of vein diameter in the total sample. Statistical analysis was performed using SAS 9.4 (SAS Institute).

## Results

**Fig 1:**
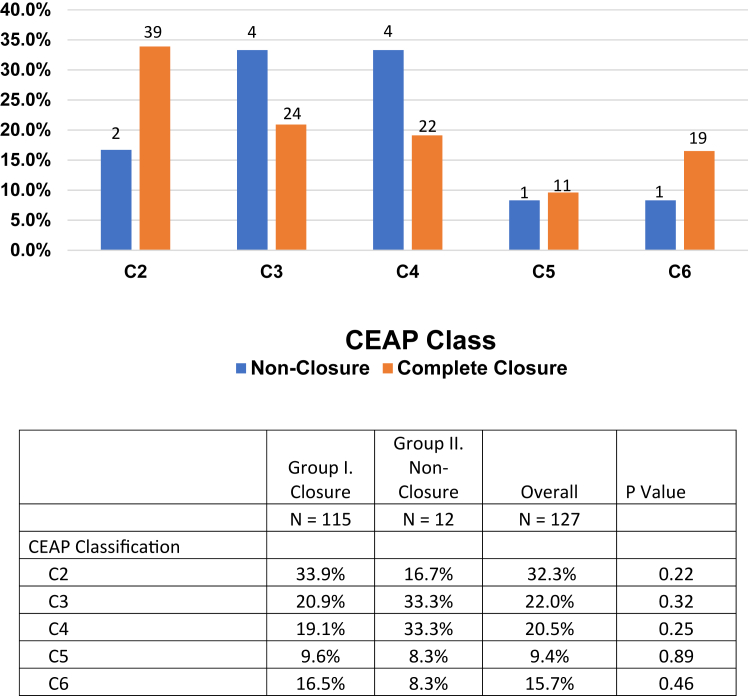
Distribution of CEAP classification.

Between June 2018 and September 2022, a total of 224 limbs underwent MFA in our ambulatory venous center. Of these, 127 limbs in 103 patients met study inclusion criteria. Complete closure (Group I) occurred in 115 limbs, and 12 limbs did not close (Group II) based on PDUS. Patient demographics for both groups can be found in Table I. The mean BMI for the entire cohort was 29.4 ± 6.4 kg/m^2^. The mean BMI in Group II (36.1 ± 6.4 kg/m^2^) was significantly higher than Group I (28.6 ± 6.1 kg/m^2^) (*P* < .001). Preoperative CEAP class distributions are reported in Fig 1. The mean VCSS for the entire cohort was 10.3 ± 3.6. There was no statistical difference in preoperative VCSS between the two groups (Group I: 10.2 ± 3.6 vs Group II: 10.8 ± 4.1; *P* = .59).

**Table I:** Patient demographics stratified by truncal vein closure

	Group I (closure)	Group II (non-closure)	Overall	*P* value
	n = 115	n = 12	n = 127	
Age, years	63.6 ± 13.6	68.4 ± 9.8	64.1 ± 13.3	.24
BMI, kg/m^2^	28.6 ± 6.1	36.1 ± 6.4	29.4 ± 6.4	**<.001**
BMI ≥30 kg/m^2^	30.4	91.7	36.2	**<.001**
Female	66.1	83.3	67.7	.22
History of hypercoagulability	1.7	0.0	1.6	.65
Deep reflux	73.9	75.0	74.0	.94
History of DVT	7.0	25.0	8.7	**.03**
On anticoagulation	9.6	16.7	10.2	.44
Reflux time, seconds	3.6 ± 1.2	3.7 ± 1.2	3.6 ± 1.2	.80

*BMI*, Body mass index; *DVT*, deep venous thrombosis.

Data are presented as percent or mean ± standard deviation.

Boldface *P* values indicate statistical significance.

Preoperative variables which were significant on univariate analysis (prior DVT, BMI ≥30 kg/m^2^, and vein diameter) were entered into a multivariate logistic regression model with the primary outcome being vein non-closure. Prior DVT was no longer significant on multivariate modeling. BMI ≥30 kg/m^2^ and vein diameter were significant by logistic regression analysis with odds ratios of 11.0 and 1.2, respectively. To identify a threshold where vein diameter is independently associated with non-closure, we dichotomized this variable. The 75th percentile of vein diameter for the overall dataset was 10 mm. A dichotomous variable of vein diameter ≥10 mm vs <10 mm was added to the model. This did not demonstrate statistical significance at a cutoff of 10 mm in the multivariable model. Vein diameter of ≥10.2 mm was independently associated with truncal vein non-closure with an odds ratio of 4.8.

Truncal veins treated included the above-knee GSV (Group I: n = 89, 77% vs Group II: n = 7, 58%; *P* = .14), below-knee GSV (Group I: n = 7, 6% vs Group II: n = 0; *P* = .38), AASV (Group I: n = 17, 15% vs Group II: n = 4, 33%; *P* = .12, and SSV (Group I: n = 4, 4% vs Group II: n = 1, 8%; *P* = .41). Two limbs in Group 1 underwent treatment of two truncal veins each (below-knee GSV/AASV and above-knee GSV/AASV). The mean truncal vein diameter treated for the entire cohort was 9.0 ± 3.9 mm. Vein size was significantly greater in Group II (14.5 ± 8.0 mm) compared with Group I (8.4 ± 2.7 mm) (*P* < .001). Superficial tributary veins were concomitantly treated with polidocanol foam in 12 patients (Group I: n = 11, 9.6% vs Group II: n = 1, 8.3%; *P* = .89). Fourteen patients (11%) underwent stab phlebectomy at a later setting. No concomitant phlebectomies were performed in this particular study cohort. Maximum tributary vein diameter was 6.5 ± 2.4 mm for the entire group (Group I: 6.4 ± 2.5 mm vs Group II: 4.8 ± 0.0 mm; *P* = .49). The overall mean foam volume was 6.2 ± 2.7 mm and not different between the two cohorts (Group I: 6.2 ± 2.6 ml vs Group II: 6.3 + 3.5 ml; *P* = .89). Operative times were similar in both cohorts (Group I: 32.5 ± 15.5 minutes vs Group II: 32.2 ± 21.5 minutes; *P* = .95).

Median follow-up was 92 days (range, 21-1350 days) in Group I and 75 days (range, 21-661 days) in Group II. Post MFA improvement in symptoms was significantly higher in Group I (96.9%) compared with Group II (66.7%) (*P* = .001). The mean postoperative VCSS was also significantly lower in Group I (8.0 ± 3.0) compared with Group II (9.9 ± 4.2) (*P* = .048). The mean change in VCSS following MFA was 2.2 ± 1.4 in Group I and 0.9 ± 0.8 in Group II (*P* = .001). Sixteen patients (13.9%) in Group I reported postoperative pain at last follow-up, and none was reported in Group II (*P* = .17). The overall incidence of postoperative superficial phlebitis was 15% (Group I: n = 18, 15.7% vs Group II: n = 10, 8.3%; *P* = .5). The ulcer healing rate for the entire cohort was 75% with no significant difference between Groups I and II (*P* = .55).

Adverse thrombotic events stratified between the two groups are summarized in Table II. The overall incidences of ARTE and DVT were 4.7% (n = 6) and 1.6% (n = 2), and all occurred in Group I. Five of the ARTEs occurred in the common femoral vein, and one occurred in the popliteal vein. Because there is no official classification system for ARTE following MFA, the American Venous Forum Endovenous Heat-Induced Thrombus (EHIT) classification system was used (EHIT II: n = 5; EHIT III: n = 1).^7^ Remote DVTs occurred in a paired femoral vein (n = 1) and in an anterior tibial vein (n = 1). Because these were not contiguous with the treated truncal vein, the EHIT classification was not applicable. There were no statistical differences noted between groups for the adverse-related thrombotic events reported. All were asymptomatic and resolved with anticoagulation (apixaban 5 mg orally twice a day). The mean duration of anticoagulation for post-MFA ARTE and DVT was 26 days. No pulmonary emboli or adverse neurologic events occurred during the study.

**Table II:** Postoperative adverse thrombotic events stratified by vein closure

Thrombotic outcomes	Group I: complete closure (n = 115)	Group II: non-closure (n = 12)	Overall (n = 127)	*P* value
Symptomatic DVT	0	0	0	1.00
ARTE	5.2	0.0	4.7	.42
Remote DVT	1.7	0.0	1.6	.65
New anticoagulation	7.0	0.0	6.3	.35
Anticoagulation, days	26.0 ± 26.9	0.0 ± 0.0	26.0 ± 26.9	.82
DVT resolved	93.0	100.0	93.7	.35

*ARTE*, Ablation-related thrombus extension; *DVT*, deep venous thrombosis.

Data are presented as percent or mean ± standard deviation.

## Discussion

Thermal ablation of incompetent superficial truncal veins is associated with excellent technical success rates and symptomatic relief.^8^ Incomplete saphenous vein closure following thermal ablation has been associated with a higher rate of symptom recurrence.^9^ Some reported risk factors for technical failure following thermal truncal vein ablation include: increased BMI, large vein diameters, anticoagulation, and male sex.^10^, ^11^, ^12^ Advantages of commercially available polidocanol microfoam over thermal ablation (RFA/laser) include significantly decreased risk of reported saphenous and/or sural nerve injuries and the ability to perform this technique without injection of tumescence.

Excellent early technical success rates following saphenous vein closure using MFA have been similarly reported. In the VANISH-2 study, a randomized, blinded, multicenter study, patients who underwent MFA with commercially manufactured 1% microfoam demonstrated greater than 77% improvement in symptoms, with 83.3% demonstrating complete occlusion or elimination of truncal vein reflux.^13^ Deak similarly reported successful early vein closure in 93.5% following 550 MFA procedures.^14^ Unlike thermal ablation, individual risk factors influencing technical failure following MFA were not reported in these studies and have not been clearly characterized in the current literature.

**Fig 2:**
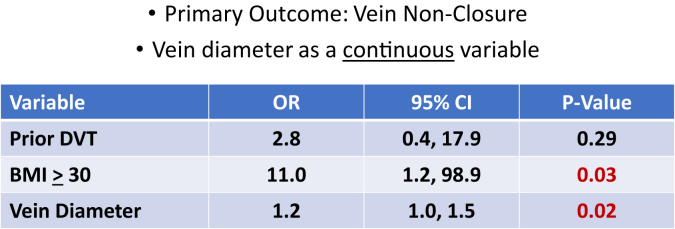
Multivariable logistic regression. *BMI*, Body mass index; *CI*, confidence interval; *DVT*, deep venous thrombosis; *OR*, odds ratio.

Our analysis demonstrated that obesity (BMI ≥30 kg/m^2^) and increased vein diameters (≥10.2 mm) were significantly predictive of truncal vein non-closure following MFA. In our study, obesity increased the odds of non-closure by 11-fold, and each millimeter increase in truncal vein diameter increased the odds of non-closure by 1.2 (Fig 2). This study also demonstrated that non-closure was significantly associated with decreased overall symptomatic relief and a smaller difference in post-MFA change in VCSS. Obesity has been found to be a risk factor for recanalization following thermal ablation, especially perforator veins.^3^^,^^15^ The results of this study are consistent with these findings following RFA and laser ablation. These pre-procedure risk factors should be considered when selecting a modality for treatment of saphenous vein reflux, and discussions with these patients should include the risk of non-closure and/or the need for repeat MFA injection to completely close the truncal vein should this occur.

Aside from these preoperative variables, Groups I and II were similar as demonstrated in Table I. Risk factors reported to be associated with worse outcomes following thermal ablation such as gender, the presence of deep venous reflux, and chronic anticoagulation were not significantly different between the closure and non-closure groups. The mean volume of microfoam used and operative times were also similar between groups.

Overall symptomatic improvement for the entire cohort (93.7%) and early closure rates (94.5%) compare favorably with outcomes reported following thermal saphenous vein ablation.^7^^,^^16^ These results were achieved despite a cohort heavily comprised with severe chronic venous insufficiency and >45% with a CEAP clinical class of 4-6 (Fig 1).

The overall incidence of adverse thrombotic events requiring a short period of post-procedure anticoagulation (ARTE: n = 6, 4.7%; DVT: n = 2, 1.6%) was 6.3%. All resolved without further clinical significance. Although this is lower than the rates reported in early randomized phase III trials which led to United States Food and Drug Administration approval for Varithena, it is higher than incidence of deep vein extension reported in the recent peer reviewed literature following thermal ablation (1.3%-1.7%).^17^, ^18^, ^19^, ^20^ The most recent clinical practice guidelines from the Society for Vascular Surgery, American Venous Forum, and American Vein and Lymphatic Society recommend against routine PDUS in asymptomatic patients to screen for ARTE and DVT following thermal ablation of the saphenous vein.^21^ Because the published incidence of ARTE is higher following MFA and optional patient selection for Varithena continues to be elucidated, we recommend early PDUS following truncal vein MFA and treatment with therapeutic anticoagulation for EHIT III and IV until the thrombus retracts caudal to the SFJ or SPJ. With this surveillance and selective treatment protocol, no clinically evident pulmonary or cerebral emboli have occurred in either our anecdotal experience with Varithena or in the randomized clinical trials leading to United States Food and Drug Administration approval.

Because of our limited follow-up and ultrasound surveillance protocol, rates of long-term recanalization and symptom relief cannot be determined from this study. The non-randomized, retrospective design of our study may also contribute to bias for patients selected for MFA over other truncal vein occlusion techniques. Quality of life measures further measuring symptomatic improvement following MFA (ie, VEINES-QOL/SYM, VVSymQ) were not used in our study. Because non-closure was a relatively infrequent event following MFA, numbers in Group II are much lower than Group I, increasing the risk of statistical error. Additionally, although 10.2 mm was the cutoff size for significance in this particular cohort, the sample size limits our ability to definitively conclude that this exact measurement is generalizable to the population.

## Conclusions

MFA of the saphenous veins results in excellent overall closure rates and symptomatic relief comparable to published outcomes following thermal ablation. Body mass index ≥30 kg/m^2^ and increased vein diameters (>10.2 mm in this particular sample) are associated with an increased risk of saphenous vein non-closure following MFA. Non-closure is also associated with overall diminished symptomatic improvement and change in preoperative and postoperative VCSS. The incidence of ARTE and DVT in this study is higher than reported rates following thermal ablation. However, MFA is safe for patients with early PDUS surveillance and selective short-term anticoagulation.

## Author Contributions

Conception and design: SF, JJ

Analysis and interpretation: ST, AC, PL, KW, SF, WD, JJ

Data collection: ST, AC, JJ

Writing the article: ST, AC, JJ

Critical revision of the article: ST, AC, PL, KW, SF, WD, JJ

Final approval of the article: ST, AC, PL, KW, SF, WD, JJ

Statistical analysis: ST, KW

Obtained funding: Not applicable

Overall responsibility: JJ

## Disclosures

J.C.J. is a consultant and speaker for Boston Scientific.
